# Ectopic Ureter Opening in Vagina: A Rare Cause of Nonfunctional Kidney and Urinary Incontinence in a Pediatric Patient

**DOI:** 10.7759/cureus.60052

**Published:** 2024-05-10

**Authors:** Abhijit Dhale, Ruturaj Pendkar, Ghanshyam Hatwar, Jay D Dharamshi, Yashasvi Trivedi

**Affiliations:** 1 Urology, Jawaharlal Nehru Medical College, Datta Meghe Institute of Higher Education and Research, Wardha, IND; 2 Surgery, Jawaharlal Nehru Medical College, Datta Meghe Institute of Higher Education and Research, Wardha, IND

**Keywords:** urinary incontinence, non functional kidney, urinary tract malformation, congenital malformation, ectopic ureter

## Abstract

An ectopic ureter is a condition characterized by a ureter, whether single or duplex, that fails to open in the trigone area of the urinary bladder but instead drains outside of it. This anomaly arises congenitally due to abnormal migration of the ureteric bud during its insertion into the urinary bladder. Here, we present a case involving an ectopic ureter draining into the vagina, with continuous urinary incontinence. We discuss the diagnosis, evaluation, and management of urinary incontinence in a female because of an ectopic ureter.

A 9-year-old girl child presented with a continuous urinary leak or incontinence requiring the use of one to two pads per day that progressively became wetter throughout the day. Physical examination revealed a normal urethral meatus and vagina without obvious visible dribbling of urine at the introitus. CT urography showed significant dilation of the right ureter, causing hydroureter and ectopic insertion of the tortuous right ureter near the external urethral orifice at the vaginal vestibule, along with an atrophic right kidney. A DTPA (diethylenetriamine pentaacetate) scan indicated the nonfunctional status of the right kidney. The patient underwent a right nephroureterectomy, leading to a complete resolution of urinary incontinence.

Ectopic ureter causing nonfunctional kidney and urinary leak or incontinence is rare. This case emphasizes the importance of a comprehensive diagnostic workup for achieving a better prognosis and initiating early treatment of ectopic ureter.

## Introduction

Ectopic ureter is a condition where the ureter fails to open in the trigone area of the bladder. Around 80% of ectopic ureter cases are linked with a completely duplex system, commonly originating from the upper pole moiety of a duplex system. The ectopic ureteric insertion may occur anywhere from the neck of the bladder to the perineum in females. The commonest site of ectopic ureter insertion is the urethra, followed by the vagina or vestibule [1]. Duplication of the ureter is a prevalent congenital urinary tract anomaly occurring more frequently in females, and it can manifest as either complete or incomplete duplication. Ectopic ureter presenting as a nonfunctional kidney and urinary incontinence is a rare finding. Urinary incontinence is characterized by the involuntary passage of any volume of urine and its prevalence increases with age. This condition can be categorized into urethral and extra-urethral types, with the latter often linked to urinary fistulas or congenital anomalies like ectopic ureter, which is exceptionally rare, occurring at an incidence of around 0.05% to 0.025% [2]. This case report describes a rare occurrence of an ectopic ureter opening into the vagina, which was observed at our institution's urology department.

## Case presentation

A 9-year-old girl child came to urology OPD with continuous urinary incontinence, necessitating the use of one to two pads per day that progressively became more saturated throughout the day. Despite this, she maintained a normal urge to urinate in between. The incontinence did not exacerbate with standing, coughing, or straining, and there were no other lower urinary tract (LUTS) symptoms, stress urinary incontinence (SUI), urinary tract infections (UTIs), fever, history of genital trauma or surgery. The patient had not yet experienced menarche. Upon physical examination, normal urethral meatus, labia, vagina, and anus were observed. There was no evident dribbling of urine at the introitus. No apparent congenital anomalies were detected. Laboratory tests, including complete blood count, revealed hemoglobin 14 gm/dl, total leucocyte count (TLC) 6700/cumm, platelets 410000 per microliter, and hematocrit 32.5% (Table 1). Renal function assessments of creatinine 1 mg/dl, urea 10 mg/dl were within normal limit (Table 2). A urine routine and microscopy examination revealed normal findings.

**Table 1:** Complete blood count (CBC) RBC: red blood cell, MCV: mean corpuscular volume, MCH: mean corpuscular hemoglobin, MCHC: mean corpuscular hemoglobin concentration, Hct: hematocrit

Test description	Result	Reference range	Unit
Hemoglobin	14	13 - 17	g/dL
Total leucocyte count	6700	4000 - 10000	/cumm
RBC indices			
RBC count	5	4.5 - 5.5	Million/cumm
MCV	88.00	81 - 101	fL
MCH	30.00	27 - 32	pg
MCHC	32.50	31.5 - 34.5	g/dL
Hct	32.5	40 - 50	%
Platelets	4.1	1.5 - 4	Lakh/microliter

**Table 2:** Kidney function test (KFT)

Test description	Result	Reference range	Unit
Urea	40	10 - 50	mg/dL
Creatinine	1.1	0.40 - 1.20	mg/dL
Sodium	180	135 - 150	mmol/L
Potassium	4	3.5 - 5.0	mmol/L

**Figure 1:**
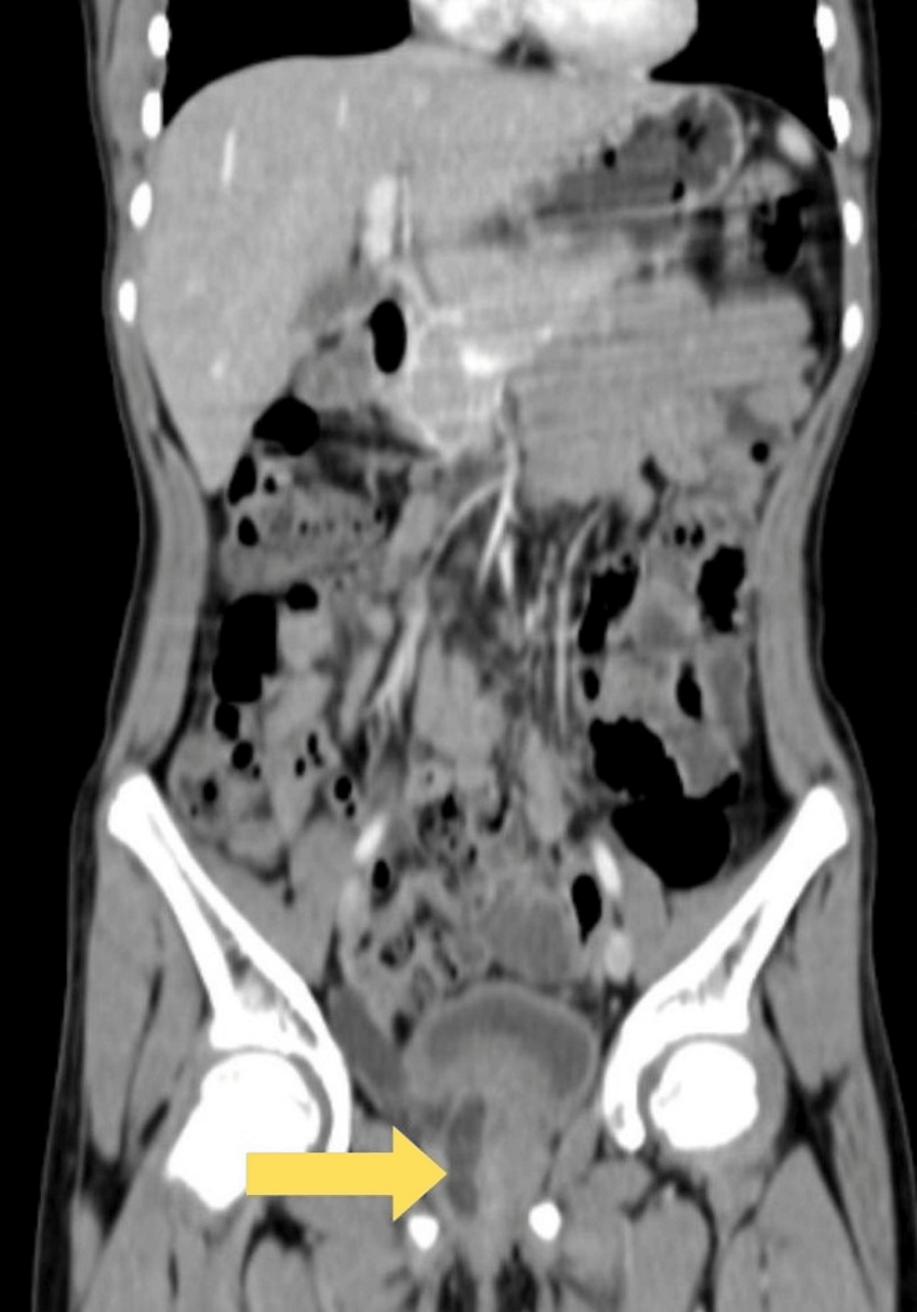
Dilated ectopic right ureter opening in the vagina Image is blurred due to motion artifact

**Figure 2:**
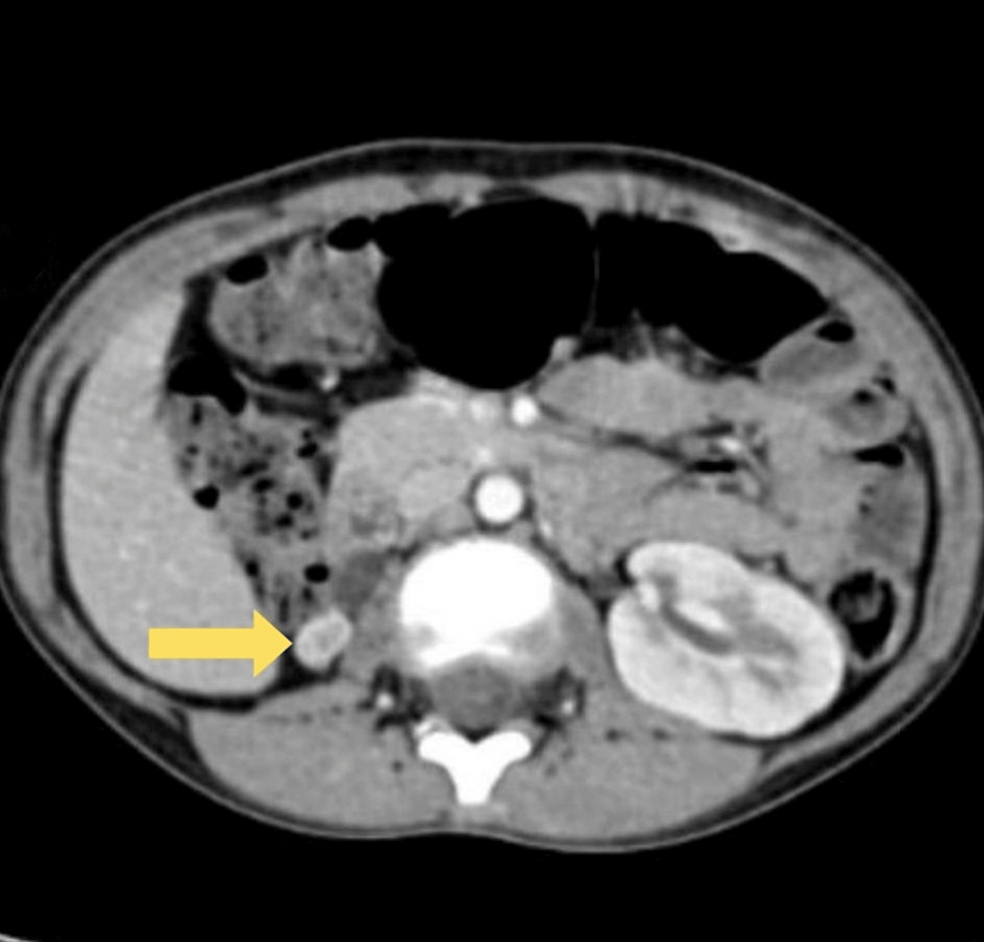
Atrophic right kidney

Imaging studies, including an ultrasound (USG) of the kidneys, ureters, and urinary bladder (KUB), showed a dilated right ureter and a small right kidney. Computed tomography urography (CT urography) further confirmed gross ureteric dilatation consistent with hydro ureter. It detected the ectopic insertion of the tortuous right ureter near the external urethral orifice at the vaginal vestibule alongside an atrophic right kidney. Conversely, the left kidney appeared normal in location, size, and shape (Figures 1, 2).

**Figure 3:**
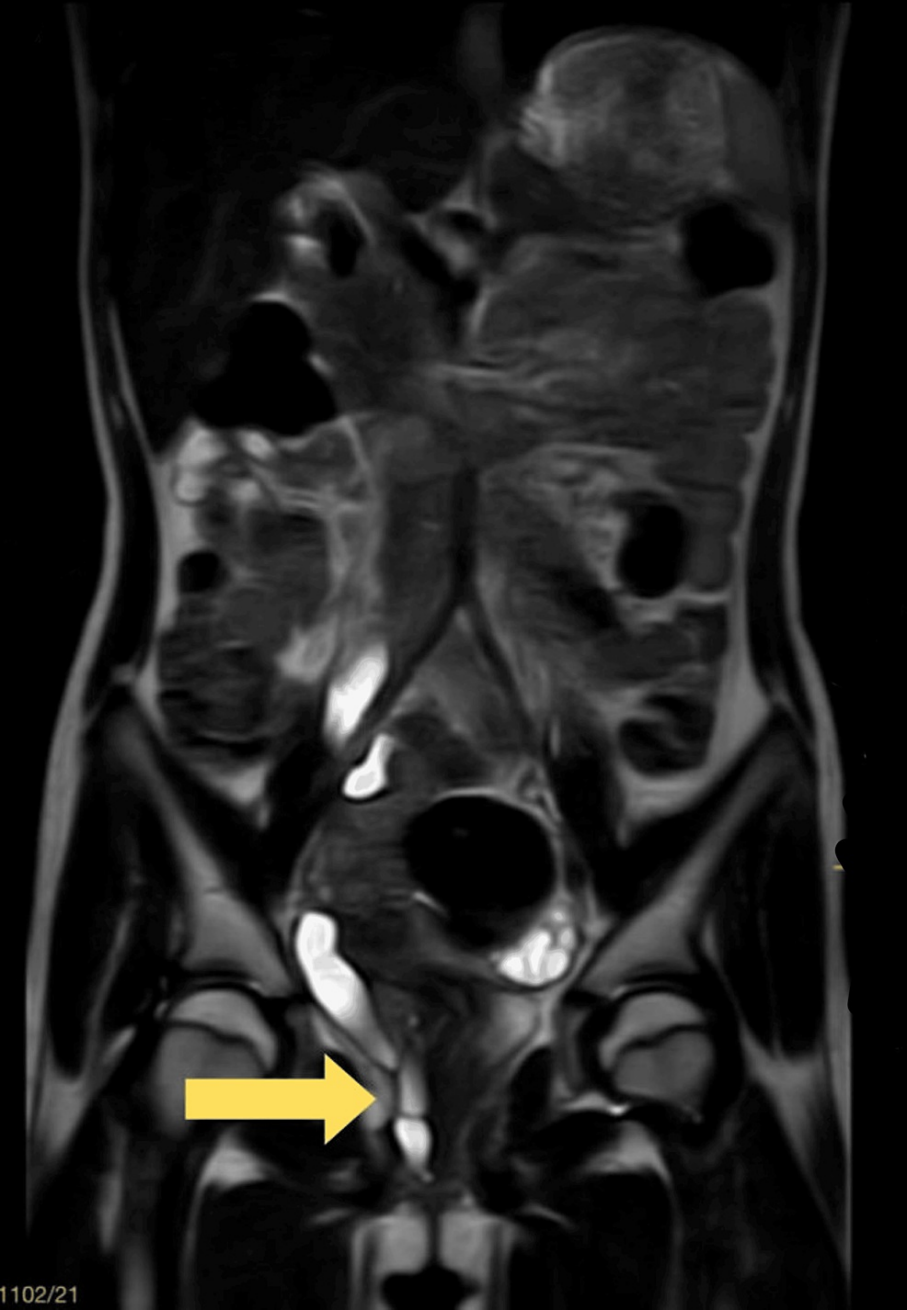
Dilated ectopic right ureter opening in the vagina

**Figure 4:**
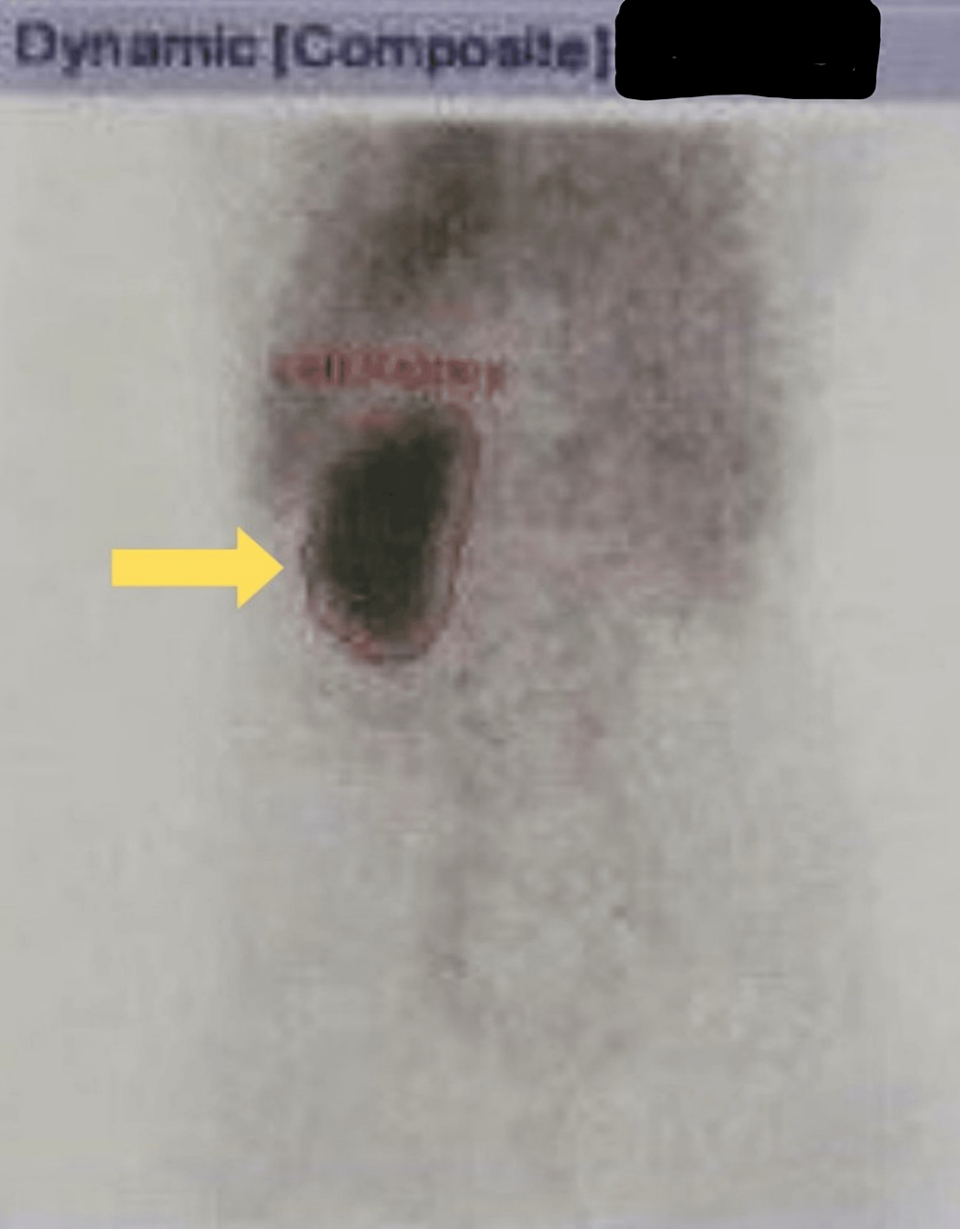
DTPA scan (showing normal left kidney) DTPA: diethylenetriamine pentaacetate

MRI pelvis revealed a dilated right ureter with abnormal ectopic insertion in the vagina (Figure 3). Additionally, a diethylenetriamine pentaacetate (DTPA) scan demonstrated non-functioning of the right kidney, with a total glomerular filtration rate (GFR) of 45.6 ml/min (Figure 4).

**Figure 5:**
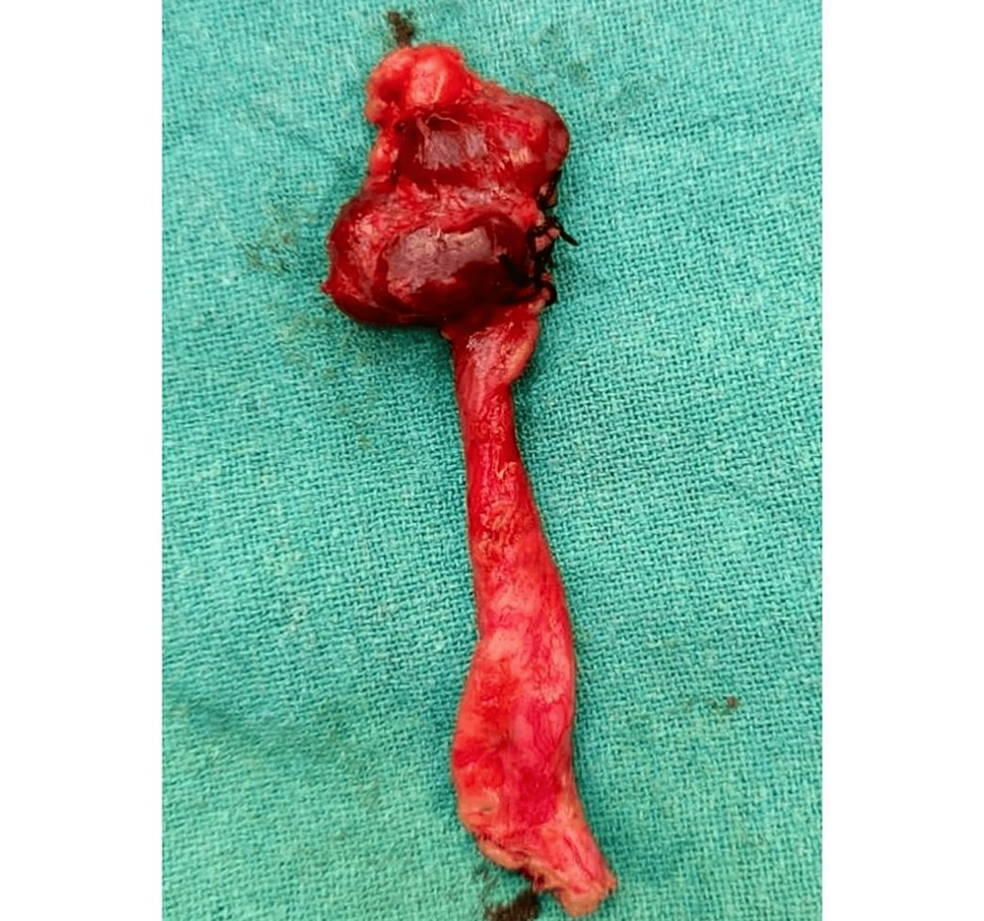
Right nephroureterectomy specimen Image by Ghanshyam Hatwar

Cystoscopic evaluations showed normal findings, except the right ureteric orifice was not visualized in the trigone region. Similarly, during vaginoscopy, no obvious visible abnormal opening of the ectopic right ureteric orifice was seen in the vagina. The patient was planned for a right nephroureterectomy. Intraoperatively, findings included an atrophic right kidney and a dilated right ureter. Retrograde ureteroscopy through the cut end of the ureter revealed a blind end of the ureter extending up to the vagina. Subsequently, laparoscopy-assisted open right nephroureterectomy (Figure 5) was performed, keeping the distal cut end of the ureter open to prevent blind loop syndrome. A postoperative single shot of antibiotic Inj. ceftriaxone as per weight given. The patient shifted to oral antibiotics. The postoperative stay was uneventful. Foley's catheter was removed on postoperative day (POD) 7. She became continent and improved symptomatically after surgery. Histopathological examination of the specimen revealed tubulointerstitial disease with chronic pyelonephritis. 

## Discussion

An ectopic ureter is described as a ureter that fails to open in the trigone region of the urinary bladder. This case brings attention to a rare congenital anomaly affecting the urogenital system, occurring in approximately one in 2000 newborns.

The overall occurrence of the ectopic ureter in the common population is approximated to range from 0.05% to 0.025%, with a higher prevalence among females, where the female-to-male ratio can be as high as 6 to 1. In approximately 80-85% of instances, the ectopic ureter is linked with a duplex renal collecting system [1]. Ectopic ureter encompasses various ureteral insertions, ranging from nearly normal to extravesical locations, resulting from abnormalities in common nephroureteric duct apoptosis or the site of ureteral bud origin. The ectopic ureter can be found anywhere from the neck of the bladder to the perineum, with the urethra being the most common site (45%), followed by the vagina (35%) and the vestibule (15%) in females. In the majority of female cases, the ureteric ectopia opens either distal to the external urethral sphincter or into the genital tract, leading to persistent urinary incontinence [3].

The patient, a 9-year-old female child, presented with a subtle yet persistent urinary leak or incontinence. Typically, girls with ectopic ureters experience continuous urinary incontinence despite maintaining a regular normal micturition pattern, as the ectopic ureter opens distal to the external urethral sphincter. Common manifestations include incontinence and recurrent UTIs. Uncommon presentations may include urine pooling at the vagina, Gartner's cyst, and hydroureteronephrotic enlarged upper pole or nonfunctional kidney [4]. Radiological imaging is crucial for confirming the diagnosis. Initially, USG KUB is often the first-line diagnostic test in children. For definitive diagnosis or exclusion of an ectopic ureter, CT urography or MRI are preferred methods. Although traditional radiological methods like ultrasound and intravenous pyelography are commonly used to evaluate the abnormal duplex system with kidneys and ectopic ureters, approximately 16% of ectopic ureteric cases may go undetected by intravenous pyelogram (IVP) because of factors such as the absent upper pole calyceal system of kidney or very thin atrophic or pyelonephritis renal parenchyma. Consequently, additional imaging techniques contrast-enhanced computed tomography (CECT), MRI, DTPA, or a dimercaptosuccinic acid (DMSA) scan are necessary [5].

A DTPA scan is necessary to assess the differential function in cases of a small, atrophic kidney or a large, hydronephrotic kidney. Surgical management of the ectopic ureter depends on the functional status of the kidney. In cases of a functioning upper pole duplex system, distal and proximal ureteroureterostomy (end-to-side) may be performed. For a nonfunctional moiety, partial nephrectomy is considered, while in the case of a small, atrophic, nonfunctional kidney, nephrectomy may be warranted. If there is an associated lower pole refluxing system, concurrent ureteric (transvesical Leadbetter-Politano or extravesical Lich-Gregoir technique) reimplantation might be necessary. In instances of a nonfunctional moiety, the risk of recurrent infections and pyelonephritis can be eliminated by conducting an upper pole partial nephrectomy or complete nephreureterectomy [3,6].

In our case, a right nephrectomy with ureterectomy was performed due to the small, atrophic nature of the right kidney resulting from improper drainage. Although ectopic ureter is a congenital anomaly that typically presents since birth, its diagnosis can be delayed until adolescence due to insufficient history-taking and assessments. As individuals age, other factors contributing to urinary incontinence are frequently given greater consideration, leading to the eventual oversight of the diagnosis of ectopic ureter [7].

Despite the availability of several major diagnostic tools, such as USG, voiding cystourethrography (MCU/RGU), IVP, and CECT, these imaging modalities often fail to provide precise information about the exact location of ectopic ureteral openings. The diagnosis of a supra-sphincteric ectopic ureteric insertion typically occurs during evaluation for the underlying cause of recurrent UTIs. Nonetheless, when the ectopic ureteric opening is infra-sphincteric, the prevailing symptom typically involves a regular urination pattern coupled with continuous urinary incontinence [8].

Sometimes, urinary incontinence may not present itself if the ectopic ureter drains an excessively atrophic renal segment or if there is compression of the lower one-third of the ureter between muscles of the perineum and external urethral sphincter, particularly until an adult or childbearing age, even when the ureter opens into the infra sphincteric region. However, symptoms may appear later in life, especially during childbirth or pregnancy, which can weaken the external sphincter [9]. In contrast, men typically remain continent since the ectopic ureter usually opens into the posterior urethra proximal to the urethral sphincter. When ectopic ureters open into the male genital tract vas epididymis or prostate, symptoms such as orchitis, epididymitis, vesiculitis, prostatitis, and blood-mixed painful ejaculation may manifest. In males, the ectopic ureter is predominantly correlated with a single collecting system, while in females, it is more commonly linked with a duplex system [10]. Furthermore, congenital anomalies like congenital heart disease (CHD), hepatic dysplasia, or renal dysplasia can coexist with ectopic ureter.

## Conclusions

The ectopic ureter opening in the vagina, resulting in a nonfunctional kidney and urinary incontinence, is an unusual occurrence. This paper delves into the approach and management of ectopic ureters in young girls. It revolves around a case wherein a young girl has been experiencing minimal yet persistent urinary incontinence since childhood due to an ectopic ureter, ultimately leading to the atrophy of her right kidney. Despite seeking assistance from various professionals, the accurate diagnosis was delayed, leading to inadequate drainage and subsequent loss of functionality in the right kidney. This delay in diagnosis and management has had significant repercussions on her quality of life and overall physical, mental, and emotional well-being. It emphasizes the necessity of a thorough diagnostic evaluation, including a detailed history, to accurately diagnose and appropriate management of ectopic ureter cases.
